# C-756-06. Standardization of Rehabilitation Consults Among Pediatric Burn Admissions

**DOI:** 10.1093/jbcr/irag033.050

**Published:** 2026-04-06

**Authors:** Alicia N Page, Rohit Mittal, Tiffany Smith, Steven A Kahn, Jenna C Kelly

**Affiliations:** South Carolina Burn Center at Medical University of South Carolina, Charleston, SC; South Carolina Burn Center at Medical University of South Carolina, Charleston, SC; South Carolina Burn Center at Medical University of South Carolina, Charleston, SC; Medical University of South Carolina, Charleston, SC; South Carolina Burn Center at Medical University of South Carolina, Charleston, SC

## Abstract

**Introduction:**

Early rehabilitation is critical for optimal functional outcomes and an expeditious recovery in burn patients. The American Burn Association requires a comprehensive rehab plan for patients within 24 hours of admission per verification standards. At our burn center, we sought to identify our baseline rates of consultation for PT and OT within 24 hours of admission and then implement measures to exceed ABA minimum rates and achieve early rehab evaluations. As part of process improvement to optimize care, a standard admission order set with mandatory rehab consultations was implemented, with an internal goal of >90% compliance. We hypothesized that this systematic approach would increase early rehab evaluations and achieve compliance with ABA verification requirements.

**Methods:**

This was a retrospective single center study from May 2023-April 2025, comparing pediatric patients who received an early (within 24 hours) consultation by PT and OT in a pre-implementation historical control group (May 2023-Feb 2024) versus a post-implementation group (March 2024-April 2025) with the enhanced order set. Data collected included admission date/time, PT consult date/time, OT consult date/time, PT/OT order acknowledgement date/time as well as gap time to consultation placement evaluated via chart review. The primary outcome was the percentage of patients receiving comprehensive rehab programs within 24 hours of admission, consistent with ABA verification criteria. Statistical analysis used Fisher's exact test, with significance set at *p*<.05.

**Results:**

Baseline analysis revealed most patients were receiving early rehab consultation. In the pre-implementation group, 77% of patients received PT and 92% of patients received OT consultations within 24 hours. Following implementation of the standardized order set, consultation rates increased significantly to 97% for PT (*p*=.0003) and 100% for OT (*p*=.014). This represents substantial improvement from already strong baseline performance to near-perfect compliance.

**Conclusions:**

Implementation of mandatory rehab consultations within a standardized pediatric burn admission order set significantly improved already strong therapy evaluation rates, achieving near-universal compliance that substantially meets ABA verification criteria and exceeds our internal target of 90%. This simple systematic enhancement optimized consultation workflows from good baseline performance to nearly perfect execution, demonstrating that straightforward process standardization can effectively address consultation variability. The intervention's effectiveness shows how process optimization can elevate high-performing systems to achieve excellence in burn care delivery.

**Applicability of Research to Practice:**

The ease of implementation and sustained results make this approach adoptable by other burn centers seeking to optimize rehab workflows and achieve the highest standards of evidence-based care.

**Funding for the study:**

N/A.

